# Physical Activity and Lipid Profile in the ELSA-*Brasil* Study

**DOI:** 10.5935/abc.20160091

**Published:** 2016-07

**Authors:** Raquel Caroline da Silva, Maria de Fátima Haueisen Sander Diniz, Sheila Alvim, Pedro Guatimosim Vidigal, Ligia Maria Giongo Fedeli, Sandhi Maria Barreto

**Affiliations:** 1 Universidade Federal de Minas Gerais, Belo Horizonte, MG - Brazil; 2 Universidade Federal da Bahia, Salvador, BA - Brazil; 3 Universidade de São Paulo, São Paulo, SP - Brazil

**Keywords:** Motor Activity, Cardiovascular Diseases, Health Profile, Hypercholesterolemia, Cholesterol, Triglycerides

## Abstract

**Background:**

Regular physical activity (PA) induces desirable changes in plasma levels of high- and low-density lipoproteins (HDL and LDL, respectively) and triglycerides (TG), important risk factors for cardiometabolic diseases. However, doubts whether intensity and duration have equivalent benefits remain.

**Objective:**

To assess the association of PA intensity and duration with HDL, LDL and TG levels.

**Methods:**

Cross-sectional study with 12,688 participants from the Brazilian Longitudinal Study of Adult Health (ELSA-Brasil) baseline, who were not on lipid-lowering medication. After adjustment for important covariates, multiple linear regression was used to assess the association of PA intensity and duration with HDL, LDL and TG (natural logarithm) levels.

**Results:**

Both moderate and vigorous PA and PA practice ≥ 150 min/week were significantly associated with higher HDL and lower TG levels. Vigorous PA was associated with lower LDL only on univariate analysis. After adjustments, moderate and vigorous PA increased mean HDL level by 0.89 mg/dL and 1.71 mg/dL, respectively, and reduced TG geometric mean by 0.98 mg/dL and 0.93 mg/dL, respectively. PA practice ≥ 150 min/week increased mean HDL level by 1.05 mg/dL, and decreased TG geometric mean by 0.98 mg/dL.

**Conclusion:**

Our findings reinforce the benefits of both PA parameters studied on HDL and TG levels, with a slight advantage for vigorous PA as compared to the recommendation based only on PA duration.

## Introduction

High plasma concentrations of low-density lipoproteins (LDL) and triglycerides (TG) and low high-density lipoprotein (HDL) levels are risk factors for cardiovascular disease (CVD).^1^ In addition to reducing the risk for CVD,^2-7^ an increase in HDL can halt the progression or even cause the regression of atherosclerotic plaques.^8^

Observational and experimental studies have shown that regular practice of physical activity (PA) induces desirable changes in plasma lipid levels,^9^ especially HDL increase and TG decrease, in addition to triggering beneficial effects on total cholesterol and its low-density and very-low-density fractions (LDL and VLDL, respectively).^10,11^ The effect of PA on HDL and TG levels seems to depend on neither weight nor diet changes.^12^ Physical activity is assumed to increase the activity of lipase lipoprotein and lecithin cholesterol acyltransferase and to reduce the activity of hepatic lipase and cholesterol esterified transfer protein, components of reverse cholesterol transport.^13^ Despite the well-known benefits resulting from PA practice, there are controversies about which PA characteristic would be more important to improve lipid profile: exercise intensity,^14^ frequency,^15,16^ duration^17^ or a combination of frequency and intensity.^14^ The reduction in TG levels was associated with higher PA intensity, but not with PA frequency.^12^

The World Health Organization (WHO) recommends, for adults aged 18-64 years, at least 150 min/week of moderate-intensity aerobic PA or 75 min/week of vigorous-intensity aerobic PA, or an equivalent combination of those, and aerobic activities should be performed in bouts of at least 10 minutes to yield benefits for cardiovascular health.^18^

Few studies in Brazil have estimated the contribution of different aspects of PA to HDL, LDL and TG concentrations. The present study was aimed at estimating the association of intensity and duration of leisure-time PA with the lipid profile of adult men and women participating in the Brazilian Longitudinal Study of Adult Health (ELSA-Brasil) and not using any lipid-lowering medication. The major objective of the ELSA-Brasil was to investigate the biological, behavioral, environmental, occupational and psychosocial determinants of the incidence of CVD and diabetes.^19,20^

## Methods

The ELSA-Brasil cohort included 15,105 active and retired individuals, aged 35 to 74 years, from teaching and research institutions in six Brazilian capitals. Because ELSA-Brasil is a multicenter study carried out in six states of three geopolitical regions of Brazil, it has an important diversity of phenotypes with information on a large number of sociodemographic, behavioral, clinical and laboratory factors that might influence lipid profile.

The present study has a cross-sectional design and uses data from the baseline of ELSA-Brasil, carried out from 2008 to 2010. In the investigation centers, data were collected by certified interviewers and checkers, meeting the criteria recommended by a Quality Control Committee,^21^ allowing the necessary standardization.

The eligibility criterion of the present study was as follows: individuals aged 35 to 69 years, undergoing laboratory tests to measure HDL, LDL and TG levels, and who answered the International Physical Activity Questionnaire (IPAQ) on leisure-time PA.

**Figure 1:**
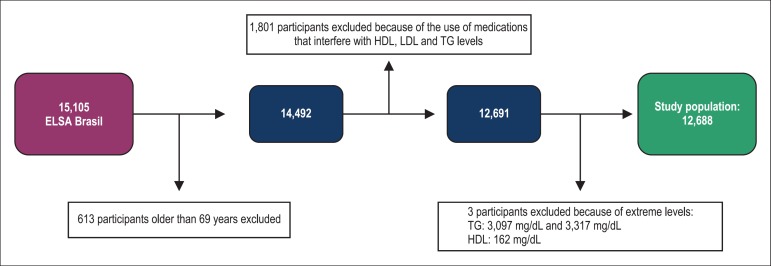
Study population selection. HDL: high-density lipoprotein; LDL: low-density lipoprotein; TG: triglycerides.

Because IPAQ was validated only for adults up to the age of 69 years, 613 participants (4.06%) aged 70 to 74 years were excluded.^22^ In addition, 1,801 participants (11.92%) were ineligible because they were on medications that could influence HDL, LDL and TG levels: atorvastatin calcium; bezafibrate; ciprofibrate; ezetimibe; fenofibrate; fluvastatin sodium; gemfibrozil; lovastatin; nicotinamide; orlistat; pravastatin sodium; simvastatin; rosuvastatin calcium; nicotinic acid; conjugated estrogens and estrone sulfate. Three individuals (0.02%) had extreme TG and HDL levels and were excluded from the analysis. The final sample comprised 12,688 individuals (Figure 1).

All laboratory parameters were measured in blood samples collected in the local investigation centers, after a mean 12-hour fasting period (minimum of 10 hours, and maximum of 14 hours). The samples were sent to the central laboratory of ELSA-Brasil in São Paulo for analysis, via a transport service specialized in frozen biological material, and processed using ADVIA 1200 automated analyzer (Siemens Healthcare Diagnostics, USA). Triglyceride levels were measured by using the colorimetric method containing glycerophosphate and peroxidase. LDL levels were estimated by using Friedewald formula, and, when TG levels were higher than 400 mg/dL, homogeneous enzymatic colorimetric assay without precipitation was used. HDL levels were measured using homogeneous enzymatic colorimetric assay without precipitation.^23^

The results of HDL, LDL and TG measurements were assessed in continuous and categorical ways. The categorical analysis was obtained from cutoff points for the desirable minimum HDL levels, ≥ 40 mg/dL for men and ≥ 50 mg/dL for women. For LDL and TG levels, the cutoff points considered were below 160 mg/dL and 150 mg/dL, respectively.^24^

Information regarding leisure-time PA practice were obtained via the long version of IPAQ for leisure-time PA, containing questions related to PA frequency, duration and intensity.^25,26^

Physical activity was categorized as follows: 1) insufficient - no PA practice OR some PA, but not meeting the other two categories; 2) moderate - 3 or more days of vigorous-intensity PA for at least 20 min/day, OR 5 or more days of moderate-intensity PA and/or walking, in combination or alone, at least 30 min/day, OR 5 or more days of any combination of walking, moderate-or-vigorous-intensity PA achieving a minimum of 600 MET-min/week; and 3) vigorous - vigorous-intensity PA on at least 3 days, accumulating a minimum of 1500 MET-min/week, or 7 or more days of any combination of walking, moderate-or-vigorous-intensity PA accumulating a minimum of 3000 MET-min/week.^26^

In addition, PA was assessed by using both the total reported PA duration in min/week, represented by the sum of the time spent with insufficient-, moderate- and vigorous-intensity PA, and the categorization into little active or active, based on the WHO recommendation of moderate PA for at least 150 min/week, or vigorous PA for at least 75 min/week.^18^

Sociodemographic characteristics included: sex; age (years); schooling (incomplete elementary, complete elementary, middle- or high-school level); per capita income obtained by dividing the mean point of each of the 10 bands of family net income per month by the number of people depending on that income (quintile); and self-reported race/skin color (black, mixed, white, yellow/native). Individuals self-reporting yellow skin color (2%) and native (1%) were grouped into a single category due to their small number.

Anthropometric indicators were body mass index (BMI), obtained by dividing body weight (kg) by square height (m^2^), and waist/hip ratio (WHR), resulting from dividing waist circumference (cm) by hip circumference (cm). Body weight and standing height were measured according to consolidated technical standards and criteria.^21^

The validated food-frequency questionnaire provided data of participants' food intake.^27^ Regarding fruits and vegetables, regular intake was defined as their usual ingestion at least five days per week, and low intake, as their usual ingestion four or less days per week. The intake of fat and carbohydrates was assessed by their daily consumption in grams.

The category "smoker" referred to having ever smoked at least 100 cigarettes during life and currently smoking, and "ex-smoker"/non-smoker referred to not currently smoking. The use of alcoholic beverages was categorized as follows: never used alcohol; ex-user; moderate user; and abuser. Alcohol abuse was defined as weekly alcohol consumption greater than 140 g and 210 g for women and men, respectively.

### Statistical analysis

The normality of distribution of each continuous variable was assessed by using Shapiro Wilk test. Variables with normal distribution were described as mean ± standard deviation, and the others, as median and interquartile intervals. The distribution of categorical variables was compared by using chi-square test, and that of continuous variables, by using Mann-Whitney test for those whose distribution was not normal. Because TG levels do not have normal distribution, they were transformed into natural logarithm (ln) in the regression model, with exponentiation of the coefficients of explanatory variables to indicate values of multiplicative changes in the geometric mean of TG levels.

The independent association of PA practice with HDL, LDL and TG (ln) concentrations was estimated by using multiple linear regression after adjusting for sex and age and the other confounding variables that remained associated with response variables to the level of p < 0.05 in the final model.

Raw and adjusted coefficients of determination (R^2^) were used to estimate the contribution of PA and other explanatory variables to the distribution of the response variables in the final model of HDL, LDL and TG. The specific percentage contribution of each different PA indicator to response-variable variability in the final model was estimated by comparing adjusted coefficients of determination before and after inclusion of the PA intensity or duration variable in the model.

The analyses were performed by using Stata statistical software, 12.0 version.

## Results

Most of the 12,688 participants included in the study were of the female sex and had white skin color and high-school level. Mean age was 50 years, mean BMI was 26.8 kg/m^2^, 15.3% smoked, and most of the participants were moderate alcohol users (Table 1).

**Table 1:** Sociodemographic, behavioral and anthropometric characteristics and lipid profile of ELSA-Brasil participants aged 35 to 69 years, according to sex (2008-2010)

**Variables**	**Male n = 5,731**	**Female n = 6,957**	**Total n = 12,688**
**Sociodemographic characteristics**			
Sex (%)	45.2	54.8	
Age in years	50.4 (± 8.2)	50.5 (± 8.1)	50.5 (± 8.1)
**Skin color/race (%)**			
White	50.5	50.5	50.5
Mixed	31.8	27.6	29.5
Black	14.6	18.2	16.6
Yellow/ Native	3.1	3.6	3.4
**Schooling (%)**			
High school	48.2	54.6	51.7
Middle school	35.5	36.7	36.1
Complete elementary	8.4	5.2	6.6
Incomplete elementary	7.9	3.6	5.6
**Behaviors**			
**Smoking (%)**			
Never smoked/ Ex-smoker	84.7	87.4	86.2
Smoker	15.3	12.6	13.8
**Alcohol consumption (%)**			
Never	4.5	15.5	10.5
Ex-user	19.9	20.1	20.0
Moderate user	63.1	60.9	61.9
Abuser	12.5	3.6	7.6
**Fruit and vegetable consumption (%)**			
≤ 4x/week	61.8	44.4	52.3
≥ 5x/week	38.2	55.6	47.7
Saturated fat intake (g)†	32.2 (23.7; 44.2)	27.4 (20.1; 36.6)	29.4 (21.5; 40.1)
Carbohydrate intake (g)†	354.6 (273.3; 468.4)	289.3 (224.6; 377.6)	316.3 (242.3; 416.9)
**Leisure-time physical activity**			
Physical activity recommended by WHO* (%)			
< 150 min/week	57.7	68.4	63.6
≥ 150 min/week	42.3	31.6	36.4
**Physical activity intensity†(%)**			
Insufficient	74.7	81.0	78.1
Moderate	14.0	12.0	12.8
Vigorous	11.3	7.0	9.1
Total physical activity duration (min/week) (median, Q1 and Q3)	80.0 (0; 240.0)	0 (0; 180.0)	60.0 (0; 190.0)
**Anthropometrics**			
Body mass index (kg/m^2^)*	26.8 (± 4.3)	26.9 (± 5.1)	26.8 (± 4.8)
Waist-hip ratio*	0.94 (± 0.07)	0.84 (± 0.07)	0.89 (± 0.09)
Lipids			
HDL (mg/dL)*	50.9 (± 12.2)	61.8 (± 14.5)	56.9 (± 14.6)
LDL (mg/dL)*	133.9 (± 35.3)	133.0 (± 34.4)	133.4 (± 34.8)
Triglycerides (mg/dL)†	131.0 (92.0; 192.0)	100.0 (73.0; 140.0)	112.0 (80.0; 163.0)

Q1 and Q3: interquartile interval; WHO: World Health Organization; (*) Duration recommended by WHO; (†) Intensity defined based on long IPAQ. HDL: high-density lipoprotein; LDL: low-density lipoprotein; (*) Mean (standard deviation); (†) Median (interquartile intervals).

Regarding weekly leisure-time PA practice, almost 80% of the individuals had it insufficient, less than 10% had it vigorous, and PA distribution was similar in both sexes. Of the participants, 64% did not report PA practice according to WHO recommendations, and the median total PA duration was 60 minutes per week for the total population (Table 1).

Regarding lipid profile, HDL and LDL values ranged from 20 to 148 mg/dL and 32 to 515 mg/dL for men, and from 18 to 146 mg/dL and 33 to 411 mg/dL for women, respectively. TG values ranged from 26 to 2,070 (mg/dL) for men and from 26 to 1,438 100 mg/dL for women (Table 1).

Less than half of the men and slightly more than half of the women met the recommendations for HDL levels, and similar prevalences were observed for desirable levels according to sex. Altered TG was observed in 3/5 of men and 2/5 of women (Table 2). Sex, age, skin color, per capita income (quintile), schooling, smoking habit, alcohol use, BMI and WHR were statistically associated with recommended HDL and TG levels. On the other hand, desirable LDL levels were statistically associated with age, schooling, smoking habit, BMI and WHR (Table 2). Recommended HDL and TG levels were statistically associated with PA intensity, total PA duration and recommended PA duration of at least 150 min/week. Desirable LDL levels were statistically associated with only total PA duration (Table 3).

**Table 2:** Distribution of study participants according to sociodemographic, behavioral and anthropometric characteristics, and according to desirable levels of HDL and LDL and triglycerides (n = 12,688)

**Variables**	**desirable** **HDL**	**altered** **HDL**	**p-value**	**desirable** **LDL**	**altered** **LDL**	**p-value**	**desirable** **TG**	**altered TG**	**p-value**
**Sex (%)**			< 0.001*			0.655*			< 0.001*
Male	46.8	37.3		45.1	45.6		38.3	61.0	
Female	53.2	62.7		54.9	54.4		61.7	39.0	
**Age (years) (%)**			< 0.001*			< 0.001*			< 0.001*
35 - 44	24.6	30.5		28.0	16.2		27.7	20.6	
45 - 54	42.4	42.7		42.2	43.7		42.1	43.3	
55 - 64	27.5	22.1		24.6	33.8		24.9	30.3	
65 - 69	5.6	4.8		5.2	6.3		5.3	5.8	
**Skin color/race (%)**			0.041*			0.440*			< 0.001*
White	50.9	49.0		50.5	50.6		50.6	50.4	
Mixed	29.0	32.0		29.3	30.2		28.6	31.6	
Black	6.8	15.7		16.7	16.3		17.5	14.4	
Yellow/Native	3.4	3.3		3.5	2.9		3.3	3.6	
**Schooling (%)**			< 0.001*			0.017*			< 0.001*
High school	52.9	45.9		51.5	52.3		54.2	45.7	
Middle school	35.1	41.1		36.6	34.2		34.9	39.0	
Complete elementary	6.5	7.5		6.6	6.9		6.1	8.0	
Incomplete elementary	5.5	5.5		5.3	6.6		4.8	7.3	
**Per capita income (%)**			< 0.001*			0.089*			< 0.001*
1st quintile (higher)	21.1	14.2		19.5	21.3		21.1	16.9	
2nd quintile	18.7	16.6		18.2	18.7		18.6	17.7	
3rd quintile	19.4	18.9		19.2	19.7		19.4	19.2	
4th quintile	21.2	24.2		22.0	20.8		21.5	22.3	
5th quintile (lower)	19.6	26.0		21.1	19.5		19.4	23.9	
**Smoking (%)**			< 0.001*			0.003*			< 0.001*
Never smoked/ Ex-smoker	86.8	83.7		86.7	84.4		87.8	82.6	
Smoker	13.2	16.3		13.3	15.6		12.2	17.4	
**Alcohol consumption (%)**			< 0.001*			0.783*			< 0.001*
Never	9.6	14.3		10.6	9.9		11.1	9.0	
Ex-user	18.7	26.2		20.0	20.0		20.2	19.6	
Moderate user	63.4	54.9		61.8	62.2		63.1	59.2	
Abuser	8.3	4.6		7.6	7.8		5.7	12.2	
**Fruit and vegetable consumption (%)**			0.783*			0.974*			< 0.001*
≤ 4x/week	52.3	52.5		52.3	52.3		50.3	56.8	
≥ 5x/week	47.8	47.5		47.7	47.7		49.7	43.2	
**BMI (kg/m^2^) (%)**			< 0.001*			< 0.001*			< 0.001*
Slimness	1.2	0.3		1.2	0.2		1.4	0.2	
Eutrophy	40.6	24.4		39.3	31.7		44.4	22.2	
Overweight	38.6	42.7		38.1	44.1		36.5	45.9	
Obesity	19.6	32.6		21.4	24.0		17.7	31.7	
**WHR (%)**			< 0.001*			< 0.001*			< 0.001*
M ≤ 0,9; W ≤ 0,85	44.7	28.6		44.1	33.4		51.8	18.8	
M > 0,9; W > 0,85	55.3	71.4		55.9	66.7		48.2	81.3	

HDL: high-density lipoprotein; desirable HDL - ≥ 40 mg/dL for men, and ≥ 50 mg/dL for women; LDL: low-density lipoprotein; desirable LDL - <160 mg/dL; TG: triglycerides; desirable TG - < 150 mg/dL; BMI: body mass index; WHR: waist-hip ratio; M: men; W: women; p < 0.001 - statistical significance;

(*) chi-square test.

**Table 3:** Prevalence of desirable levels of HLD, LDL and triglycerides according to indicators of physical activity intensity and duration of ELSA-Brasil participants aged 35 to 69 years, 2008-2010, n = 12,688

**Variables**	**desirable** **HDL**	**altered** **HDL**	**p-value**	**desirable** **LDL**	**altered** **LDL**	**p-value**	**desirable** **TG**	**altered TG**	**p-value**
**PA (WHO) (%)**			< 0.001*			0.391*			< 0.001*
< 150 min/week	61.8	71.8		63.8	62.9		62.2	66.9	
≥ 150 min/week	38.2	28.2		36.2	37.1		37.9	33.1	
**PA intensity (%)**			< 0.001*			0.046*			< 0.001*
Insufficient	76.9	83.7		78.3	77.7		76.6	81.7	
Moderate	13.3	10.7		12.5	14.1		13.5	11.3	
Vigorous	9.8	5.7		9.3	8.6		9.9	7.1	
Total PA duration (min/week)‡	60.0	0.0	< 0.001†	60.0	0.0	< 0.001†	60.0	30.0	< 0.001†

HDL: high-density lipoprotein; desirable HDL - ≥ 40 mg/dL for men and ≥ 50 mg/dL for women; LDL: low-density lipoprotein; desirable LDL - ≤160 mg/dL; TG: triglycerides; desirable TG - ≤ 150mg/dL; p < 0.001 - statistical significance; WHO: World Health Organization; PA: physical activity;

(*) chi-square test;

(†) Wilcoxon test (Mann-Whitney);

(‡) Median.

Table 4 shows the results of simple linear regression analysis. Moderate- and vigorous-intensity PA was associated with an increase in HDL, with suggestion of dose-response gradient. Both longer total PA duration and PA practice for at least 150 min/week or more were associated with HDL elevation. All associations maintained after adjustment for sex and age, and later for the other confounding variables including race/skin color, per capita income, schooling, WHR, BMI, alcohol consumption, current smoking and total carbohydrate and saturated fat intake (Table 4). The regular consumption of fruits and vegetables was not statistically associated with HDL levels on multivariate regression analysis.

**Table 4:** Association of different indicators of leisure-time physical activity with HDL, LDL and triglyceride levels on simple linear and multiple regression analyses of ELSA-Brasil participants (n = 12,688)

	**Univariate**			**Adjustment for sex and age‡**			**Adjustment for all confounding factors§**		
**Variables**	**β**	**[95%CI]**	**R^2^**	**B**	**[95%CI]**	**Adj R^2^**	**β**	**[95%CI]**	**Adj R^2^**
**HDL**									
PA intensity			0.003			0.152			0.266
Moderate	1.592	(0.822; 2.361)*		2.087	(1.376; 2.799)*		0.893	(0.219; 1.566)†	
Vigorous	2.265	(1.368; 3.162)*		3.927	(3.097; 4.758)*		1.710	(0.918; 2.501)*	
PA (WHO)			0.003			0.153			
≥ 150 min/week	1.522	(0.996; 2.048)*		2.772	(2.284; 3.260)*		1.055	(0.582; 1.527)*	0.266
Total PA duration (min/week)	0.004	(0.003; 0.005)*	0.002	0.007	(0.006; 0.008)*	0.153	0.003	(0.002; 0.005)*	0.267
**TG (ln)**									
PA intensity			0.006			0.093			0.230
Moderate	-0.044	(-0.072; -0.016)*		-0.073	(-0.097; -0.046)*		-0.020	(-0.045; 0.005)	
Vigorous	-0.136	(-0.168; -0.103)*		-0.163	(-0.195; -0.132)*		-0.068	(-0.097; -0.039)*	
PA (WHO)						0.091			0.229
≥ 150 min/week	-0.058	(-0.077; -0.038)*	0.003	-0.093	(-0.111; -0.074)*		-0.022	(-0.040; -0.005)†	
Total PA duration (min/week)	0.000	(0.000; 0.000)*	0.003	0.000	(0.000; 0.000)*	0.092	0.000	(0.000; 0.000)*	0.230
**LDL**									
PA intensity			0.001			0.025			
Moderate	1.564	(-0.279; 3.407)		0.609	(-1.215; 2.433)				
Vigorous	-2.282	(-4.430; -0.134)†		-1.501	(-3.631; 0.628)				
**PA (WHO)**									
≥ 150 min/week	0.307	(-0.952; 1.567)	0.000						
Total PA duration (min/week)	-0.001	(-0.004; 0.002)	0.000						

B: β coefficient; CI: confidence interval; R^2^: coefficient of determination; AdjR^2^: adjusted coefficient of determination; HDL: high-density lipoprotein; LDL: low-density lipoprotein; TG: triglycerides; PA: physical activity; PA intensity: intensity of PA defined based on short IPAQ; TG: triglyceride; WHO: World Health Organization; Total PA duration: sum of PA in minutes per week;

(*) Statistical significance p < 0.001;

(†) Statistical significance 0.05 > p > 0.001;

(‡) Multiple regression with HDL, LDL and TG (natural logarithm) adjusted for sex, age;

§ Multiple regression with HDL and TG (natural logarithm) and PA adjusted for sex (only HDL), age, skin color, schooling (only HDL), per capita income, alcohol consumption, smoking, waist-hip ratio, body mass index, total carbohydrate and saturated fat intakes.

On multiple linear regression, comparing the adjusted coefficient of determination before and after the inclusion of the variable 'PA intensity' (R^2^ = 0.265 and 0.266, respectively), PA intensity explains only 0.1% of the total variability of HDL levels in the population studied. On the other hand, PA duration ≥ 150 min/week and total PA duration explained, respectively, 0.1% and 0.2% of the total variability of HDL levels in the ELSA-Brasil cohort, considering that adjusted coefficient of determination after the inclusion of those two PA variables were 0.266 and 0.267, respectively.

Comparing to insufficient-intensity PA, moderate-intensity PA was associated with a 0.89-mg/dL increase in HDL levels, and vigorous-intensity PA was associated with a 1.71-mg/dL increase in HDL levels, after adjusting for confounding factors (Table 4). That is, individuals with insufficient PA and HDL level of 50.8 mg/dL could have their HDL increased to 51.7 mg/dL or 52.5 mg/dL, when practicing moderate- or vigorous-intensity PA, respectively, and maintaining all other characteristics, behaviors and measures unchanged.

On simple linear regression, higher PA intensity, PA practice of at least 150 min/week and total PA duration (min/week) were associated with lower TG levels (Table 3). The associations found remained statistically significant after adjusting for sex, age and other confounding variables (race/skin color, per capita income, WHR, alcohol consumption, current smoking, and total carbohydrate and saturated fat intake).

The final adjusted results show that, as compared to insufficient PA, moderate and vigorous PA associated with a 0.98-mg/dL and 0.93-mg/dL reduction in the geometric mean of TG, respectively, indicating dose-response relationship. This implies that individuals with TG levels of 111.9 mg/dL and insufficient PA could reduce their TG values to 109.7 mg/dL or 104.6 mg/dL, respectively, by performing moderate or vigorous PA, and maintaining all other behavioral and anthropometric factors unchanged.

A PA practice greater than 150 min/week, as recommended by WHO, associated with a 0.98-mg/dL reduction in the geometric mean of TG. The comparison of the adjusted coefficient of determination of multivariate analysis before and after the inclusion of the 'PA intensity' variable (R^2^ = 0.228 and 0.230, respectively) indicates that PA explains only 0.2% of the variability of TG levels in the population studied. On the other hand, according to WHO recommendation, PA explains 0.1% of that distribution, because the adjusted coefficient of determination after the inclusion of PA intensity was 0.229.

On univariate analysis, LDL showed a statistically significant association with only vigorous-intensity PA. However, after adjusting for sex and age, PA association with LDL levels lost statistical significance; therefore, multivariate analysis was not performed (Table 3).

## Discussion

The present study showed that PA practice, categorized into different aspects, associated independently with higher HDL concentrations and lower TG levels in a large sample of individuals not using lipid-lowering drugs. In addition, our results do not indicate that PA intensity is clearly superior or inferior to the PA duration of 150 min/week recommended by WHO. Considering that the Brazilian population has significant cardiovascular morbidity and mortality and high sedentary rate, our results are relevant because they emphasize the need to maintain and widen public programs to foster regular PA practice, aimed at improving the population's lipid profile.

Our results support those reported in studies assessing HDL and TG. Similarly, in studies on physical training, HDL increase and TG reduction are more frequently observed than a reduction in total cholesterol or LDL levels.^28^

To assess the impact of PA duration and intensity on HDL, LDL and TG levels, we analyzed separately the different parameters of PA classification. Some studies have reported a dose-response relationship in the association between PA intensity and lipid profile improvement, mainly HDL elevation and TG reduction in previously sedentary or inactive populations.^6,9-11,29-31^ In accordance with that, we found that the greater the PA intensity, the higher the mean increase in HDL levels and the lower the TG levels.

Three meta-analyses on the impact of physical training have shown mean increases in HDL of 1.2, 2.53 and 1.95 mg/dL, and mean reductions in TG of 15.8 and 7.12 mg/dL after the intervention.^17,32,33^ We found a mean increase in HDL of 0.89 mg/dL in individuals practicing moderate PA as compared to insufficient PA, and a 1.71-mg/dL increase for vigorous PA. Moderate PA reduced by 0.98 mg/dL the geometric mean of TG as compared to insufficient PA, while vigorous PA reduced by 0.93 mg/dL. The lipid profile improvement has a direct impact on the risk for CVD and diabetes. The *Lipid Research Clinic Prevalence Mortality Follow-up* (LRCF) has shown that the 1-mg/dL increase in HDL reduced by 3.5% the risk of coronary artery disease (CAD), and by 3.7% and 4.7% mortality in men and women, respectively.^34^ According to the results of HEART, a 1-mg/dL increment in plasma HDL is associated with a 2% to 3% reduction in the risk for CAD.^7^

Unlike studies reporting that the amount of exercise, as compared to exercise intensity, determined a higher difference in plasma lipoprotein concentrations,^15,35,36^ the present study found that both frequency and intensity are important for that effect, intensity exerting greater interference. Considering the findings of the ELSA-Brasil study and controversies in the literature, it is necessary to clarify the role played by PA, especially regarding PA amount and intensity, to improve the PA recommendations aimed at increasing HDL and reducing TG.

A Dutch cross-sectional study with individuals of three ethnicities, using the SQUASH questionnaire to measure PA level and the same ELSA-Brasil criteria to define favorable lipid profile, has reported that PA intensity, but not PA duration, associated with a more favorable lipid profile. Total PA duration associated with better HDL and TG levels only in Afro-Surinamese individuals.^11^ We observed a stronger association between vigorous-intensity PA and favorable lipid profile, in addition to an association with total PA duration per week, regardless of PA intensity. It is worth noting that 25% of the individuals had no leisure-time PA (total leisure-time PA = 0). Our results suggest that vigorous PA, but not moderate PA, is associated with greater benefit to lipid profile as compared to PA duration. It is worth noting that because of the cross-sectional design of this study, a causality relationship between PA, in any modality assessed, and TG and HDL levels could not be inferred.

According to 2011 WHO recommendation, adults should practice at least 150 min/week of insufficient-to-moderate-intensity PA during leisure time or at least 75 min/week of vigorous PA. In the present study, 64% of the participants did not reach the recommended PA level during leisure time, that percentage being higher among women (68.4%) than among men (57.7%). In addition, we observed that PA practice during the time recommended by WHO was associated with significantly higher HDL levels and lower TG levels, regardless of intensity. Therefore, our results support the WHO recommendation of at least 150 min/week of PA, which is simpler and easier to be disseminated as compared to intensity-based recommendations.

This study design observed measures to assure the quality of the information. The collection of data and biological material in the six centers followed strictly standardized procedures, undergoing constant quality control. Storage and laboratory tests were centralized in a certified laboratory. The sample was sufficiently large and heterogeneous (sex, age, schooling and behaviors) to assure statistical power for the analyses performed.^23^

It is worth noting, however, that the leisure-time PA section of the long IPAQ questionnaire has limitations and lower accuracy than PA measurement by use of objective devices.^37,38^ In large epidemiological investigations, such as ELSA-Brasil, however, the use of a questionnaire is an easy and low-cost way to assess PA, providing information that allows estimating PA levels, intensity and frequency. In addition, IPAQ has been validated in several countries, including in Brazil.^39,40^

The present study estimated the independent contribution of leisure-time PA during the past week on current HDL, LDL and TG levels. Part of the individuals classified as active in the past week might not be active routinely and vice-versa, or non-active individuals in the past week might be active most of the time. However, those errors might not be differential concerning lipid profile. Non-differential errors tend to attenuate the estimated associations, leading to underestimation of the real PA contribution to lipid profile in this study. It is worth noting that our analysis considered PA during neither transportation nor occupation.

## Conclusion

A beneficial association exists between higher PA levels and a favorable HDL and TG lipid profile for men and women. Vigorous, but not moderate, PA practice was associated with more positive lipid profile changes than PA duration alone. Such findings contribute to support public policies of prevention and reduction of the risk for cardiometabolic diseases based on the expansion and facilitation of leisure-time PA practice.
